# Toward Standardizing a Lexicon of Infectious Disease Modeling Terms

**DOI:** 10.3389/fpubh.2016.00213

**Published:** 2016-09-28

**Authors:** Rachael Milwid, Andreea Steriu, Julien Arino, Jane Heffernan, Ayaz Hyder, Dena Schanzer, Emma Gardner, Margaret Haworth-Brockman, Harpa Isfeld-Kiely, Joanne M. Langley, Seyed M. Moghadas

**Affiliations:** ^1^Department of Population Medicine, University of Guelph, Guelph, ON, Canada; ^2^International Programmes, London School of Hygiene and Tropical Medicine, University of London, London, UK; ^3^Department of Mathematics, Centre for Disease Modelling, The University of Manitoba, Winnipeg, MB, Canada; ^4^Department of Mathematics and Statistics, Centre for Disease Modelling, York University, Toronto, ON, Canada; ^5^Division of Environmental Health Sciences, College of Public Health, The Ohio State University, Columbus, OH, USA; ^6^Public Health Agency of Canada, Ottawa, ON, Canada; ^7^National Collaborating Centre for Infectious Diseases, The University of Manitoba, Winnipeg, MB, Canada; ^8^Canadian Center for Vaccinology, Dalhousie University, IWK Health Centre and Nova Scotia Health Authority, Halifax, NS, Canada; ^9^Agent-Based Modelling Laboratory, York University, Toronto, ON, Canada

**Keywords:** infectious disease modeling, reproduction number, community of practice, lexicon of terms, public health

## Abstract

Disease modeling is increasingly being used to evaluate the effect of health intervention strategies, particularly for infectious diseases. However, the utility and application of such models are hampered by the inconsistent use of infectious disease modeling terms between and within disciplines. We sought to standardize the lexicon of infectious disease modeling terms and develop a glossary of terms commonly used in describing models’ assumptions, parameters, variables, and outcomes. We combined a comprehensive literature review of relevant terms with an online forum discussion in a virtual community of practice, mod4PH (Modeling for Public Health). Using a convergent discussion process and consensus amongst the members of mod4PH, a glossary of terms was developed as an online resource. We anticipate that the glossary will improve inter- and intradisciplinary communication and will result in a greater uptake and understanding of disease modeling outcomes in heath policy decision-making. We highlight the role of the mod4PH community of practice and the methodologies used in this endeavor to link theory, policy, and practice in the public health domain.

## Introduction

Mathematical and computational models are useful tools to provide important information on key aspects of infectious disease epidemiology. Models can assist in quantifying the transmission potential of a pathogen within a population, as well as assessing the effects of various control and prevention strategies. In times of uncertainty, public health responses may be based on expected outcomes obtained from models (1), especially in the event of new emerging or re-emerging diseases. However, such outcomes are reliant on assumptions, terms, parameters, and their definitions and inter-relations (2). Models are also updated over time as empiric data accumulates, incorporating biological, epidemiological, and immunological data, as well as more realistic assumptions with the inclusion of individual-level health and behavioral responses. The use of modeling by public health decision makers is still limited, perhaps due in large part to the inconsistent use of modeling and disease-related terminology across models and disciplines (3). For example, a recent review of influenza modeling literature highlighted the discrepancies in the definition of terms used in different studies (4). Such discrepancies often arise from a model’s assumptions, methodological approaches used to analyze a model, and outdated definitions that do not reflect current knowledge.

Ambiguities caused by inconsistent use of terms in models can have enormous impact on model design, usefulness for public health, and understanding and comparability of model outcomes. Standardization of the terms used in modeling could improve this process and its applications. In recent years, considerable efforts have been made to develop unity of thought about modeling approaches in health sciences, including the particular case of infectious diseases (5). For example, there have been efforts to improve the use of disease modeling terminology through the creation of topic specific glossaries (6, 7). While such glossaries define commonly used terms and provide an understanding of the vocabulary and methods used in the disease modeling literature (3, 4, 6, 7), they do not aim at standardizing a common lexicon based on infectious disease terminology. Furthermore, the existing modeling glossaries do not take into account the spectrum of definitions of terms used by various health disciplines such as public health, epidemiology, and clinical medicine.

To address this gap, we developed a multidisciplinary dialog between the members of a recently established virtual “Community of Practice,” called mod4PH (Modeling for Public Health) (8), whose goal is to enhance the understanding of modeling, and its applications for public health and clinical decision-making related to infectious disease prevention and control. The mod4PH group, a LinkedIn forum, consists of individuals from different disciplines including disease modeling, public health, infectious disease epidemiology, and policy analysis. The objective of the current study in the mod4PH group was to produce a “glossary of terms,” which describes the standard use and definition of infectious disease modeling terms. This glossary and the guidelines for the use of terms were developed based on literature review, online discussion among mod4PH participants, and subsequent consensus. Here, we discuss the process by which the glossary was created and provide a summary of the online forum discussions surrounding the usage and definition of key terms in modeling infectious diseases.

## Materials and Methods

A comprehensive literature review of infectious disease terms used in modeling studies was combined with an online discussion in “mod4PH,” a predominately social media platform complemented by a face-to-face annual meeting. Considering the importance of a standard lexicon of terms in understanding modeling outcomes and their comparability, we discussed several key terms that are commonly used in disease models.

### mod4PH Community of Practice

Sponsored by the National Collaborating Center for Infectious Diseases (NCCID), Canada, mod4PH represents a virtual community of practice that promotes the best procedures for research activities and collaborations and integrates resources and expertise for knowledge translation to improve the uptake of infectious disease modeling (8). The mod4PH community of practice currently hosts members from various disciplines and geographic locations and recruits individuals with the required expertise and experience relevant to its mandate.

The mod4PH forum was initially established with a number of participants from the face-to-face 2014 Pan-InfORM workshop (9). Following the inception of mod4PH, NCCID announced the forum on Twitter, electronic mail, and other media in order to reach out to modelers and medical and public health professionals to enhance the depth and breadth of expertise in the forum. At the time of this study, there were 77 mod4PH members of whom approximately 50% were modelers, and the remaining were from other relevant disciplines as indicated by information included in their LinkedIn profiles. The majority of mod4PH members are located in North America (Canada and the United States). Members from Western and Southern Europe, South Central Asia, South America, and Australia and New Zealand constitute approximately one-fifth of the forum.

### Development of Common Lexicon

The literature review was conducted for specific terms that are used in peer-reviewed published articles in a wide array of journals published in English. The search engines “PubMed,” “Google Scholar,” “Web of Science,” and “Scopus” were used to find terms used in both mathematical epidemiology and public health with an ambiguous and discrepant definition. We also consulted a previous review of terms in modeling influenza infection, which relied on a variety of sources including systematic reviews, peer-reviewed published articles, books, advisory health reports, and websites of public health agencies and organizations (e.g., World Health Organization, U.S. Centers for Disease Prevention and Control, Public Health Agency of Canada, and European Center for Disease Control) (4).

We considered essential terms that are used often in epidemiological models of infectious diseases with two main criteria: (i) a term was defined differently between articles or (ii) two different terms were used interchangeably, with the threshold that one of the criteria is met in at least two peer-reviewed articles. Terms and definitions identified in the review of relevant studies were classified as “discussion topics” based on their definitions and usage. Each week, a discussion topic and the associated references were posted on the mod4PH forum (8), which remained active for the period of study between November 2015 and April 2016. The topics typically opened with a question or comment on inconsistent terms that would draw members of the forum with relevant expertise into the discussion. A total of 25 terms were discussed in 13 discussion sessions (10).

### Forum Discussion

For the purpose of this study, a convergent discussion process was followed (11), which involved a technique that allowed participants to not only provide feedback on the discussion topic but also propose questions with the flexibility to probe and explore emerging issues in different contexts (e.g., epidemiological, clinical, public health, modeling, and specific disease or population). Convergence was achieved by asking probing questions that became progressively more detailed and specific in order to clarify the definitions and appropriate use of terms in models. The general nature of questions and ensuing discussions led the forum participants to highlight the relevance of each term to the conceptual modeling frameworks, identify the most commonly used lexicon with reference to published studies in different disciplines, and challenge, change, or confirm emerging interpretations to develop a glossary of terms that can be used to standardize the vocabulary in models. New topics were introduced each week and remained open for discussion for the duration of the project to: (i) increase understanding and clarity of modeling terms for their definition and use, (ii) challenge emerging issues in closely relevant concepts and terminologies of infectious diseases, and (iii) ensure that the study of terms was not prematurely closed.

## Modeling Terms, Parameters, and Their Relations

Models of infectious disease dynamics are developed to reflect: (i) the biology of the infectious agent and (ii) the physiological processes and attributes of the disease at both the individual and population levels. These are defined by: (a) the time course of the stages of disease progression through the infection process, from exposure to recovery or death (which constitutes the area of clinical medicine) and (b) a time course of states of transmission potential from exposure to post infectiousness (which constitutes the area of public health and epidemiology) (12). The biology of the infectious agents and the pathophysiological processes include the disease statuses of the individuals, which determine the susceptibility of individuals to infection or transmissibility of the disease. The change of status described in models is often linked to the spread of disease characterized by the population-level phenomena of disease incidence or prevalence, as well as parameters affecting these phenomena such as the generation interval and serial interval. In the following sections, we report on the outcomes of discussions from the online mod4PH forum on the relevant modeling terms.

### Compartmental Models

**Figure 1:**
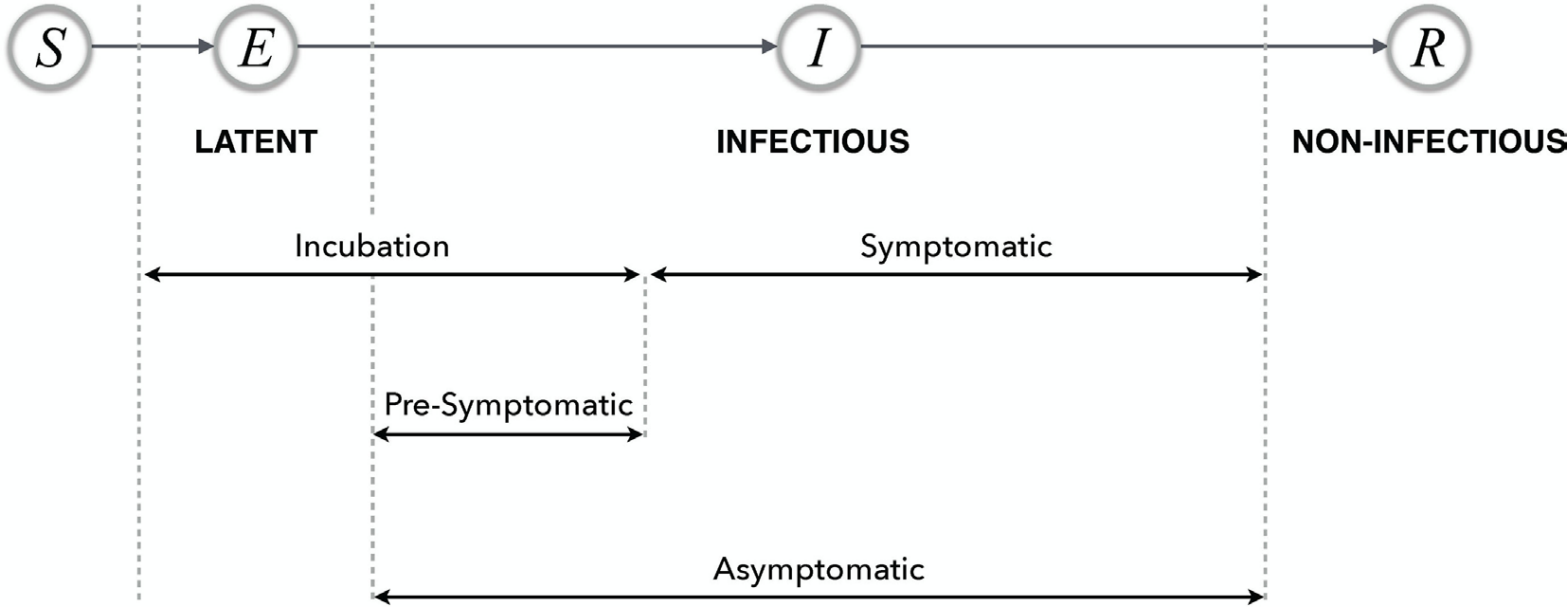
**Possible timelines and various stages of disease with their relationship in the SEIR model**. The terms latent, infectious, and non-infectious are transmission-related, and the remaining terms are all progression-related.

In most disease dynamic models, a compartmental structure is developed that divides the population into several classes of individuals according to their epidemiological statuses. These include: susceptible (S), exposed (E), infectious (I), and recovered (R), and their relationship describes a basic disease transmission dynamic model, referred to as the classical SEIR model (Figure 1) (13). Although other context and disease-specific compartments may be added, in this paper, we restrict our attention to this basic framework. Individuals may transition between these classes as a change of status occurs due to disease-related processes. These models may be used to compute quantities such as the basic reproduction number and the prevalence and incidence of disease in the population (13).

### Susceptible, Infected, and Recovered

Susceptible refers to a non-infected individual (or population) who may become infected through contact with individuals or environmental organisms that can transmit the disease. Individuals may show varying degrees of susceptibility based on a number of host factors (e.g., immunity; see “Individual Immunity and Herd Immunity”). Successful transmission of the pathogen to a susceptible individual will result in the susceptible individual becoming infected, either clinically or subclinically. However, even in the absence of successful transmission, the susceptible individual is referred to as “exposed” (as used in epidemiology and other health-related disciplines). In contrast, in compartmental models of disease transmission, the exposed compartment is used to label individuals as “infected,” with the assumption of successful transmission. It is assumed that individuals in the exposed class are incapable of transmitting the disease, unlike those in the infectious compartment.

In disease dynamic models, infected individuals are often considered to be infectious, that is, they are capable of transmitting the disease. When this classification is made, it is important for the model to clearly state that these individuals are both “infected and infectious” since the assumption of infectivity is inconsistent with the epidemiological observation that an infected individual is not necessarily infectious.

For a disease in which the causative pathogen can be eliminated from the infected person or become dormant, the infectious stage is followed by a non-infectious stage. Models of disease transmission dynamics refer to this non-infectious stage as “recovered,” since the individuals in this stage can no longer transmit the disease. However, we note that, in the epidemiological and clinical contexts, a non-infectious individual is not necessarily pathogen-free.

### Exposed and Latent

In most disease transmission dynamic models, the term “exposed” is used to refer to individuals who are infected but are not yet capable of transmitting the disease (14, 15). However, this concept is inconsistent with the observation used in epidemiology referring to susceptible individuals who have been in contact with infectious individuals, but the success of transmission is not determined. When the exposure to the infectious individual occurs with successful pathogen transmission to a susceptible individual, this leads to colonization or infection. To improve a model that includes an exposed compartment, it is suggested to use the term “latent” to represent the class of individuals who have been infected following exposure to disease but are not yet capable of transmitting the disease.

The term “latent” describes an infected individual who cannot transmit the pathogen, regardless of the time for exposure to the pathogen. For a number of infectious diseases (e.g., influenza), the individual becomes latent immediately following the exposure to pathogen when transmission occurs (14, 15). However, it is important to note that an individual may be latent at a different stage of disease. For example, individuals with tuberculosis can have active infection in which they shed bacteria or latent infection (possibly after an effective course of treatment) in which there are no clinical symptoms and no pathogen transmission, but the bacteria is still present in the body (16, 17). We note that, in clinical infectious diseases, latency can occur before symptoms, without symptoms, or after symptoms.

### Periods: Latent, Incubation, and Infectious

The latent period refers to the period of time between exposure to a disease with successful transmission and the onset of infectiousness. During the latent period, infected individuals do not transmit the disease. This period has commonly been referred to as the “exposed period” in infectious disease modeling; however, the exposed period may suggest a period of time during which the event of exposure to disease continually occurs, rather than a period of time following exposure. It is therefore suggested that the “latent period” should be used as the standard lexicon in models of disease transmission.

The incubation period is defined as the period of time between exposure to the disease (if transmission occurs) and the onset of clinical symptoms. For diseases in which the onset of infectiousness coincides with the appearance of symptoms, the latent and incubation periods are the same. However, in a number of diseases (e.g., influenza, measles, and varicella), the infected individual may become infectious before the onset of symptoms. For such diseases, the models may account for the incubation period by including the latent and presymptomatic periods (see “Presymptomatic and Asymptomatic”).

The infectious period is defined as the time interval in which the infected individual is capable of transmitting the disease. Since this period may overlap with the incubation period, it may be difficult to obtain accurate estimates of the infectious period.

### Incidence and Prevalence

Disease incidence is defined by both epidemiologists and modelers as the number of new cases in a population generated within a certain time period. In SEIR models, it is often calculated as the product of the number of susceptible individuals, the number of infected individuals (who are considered infectious and can transmit the disease), and the transmission rate per contact per unit of time. In continuous time models, the incidence of a disease is measured at a single time point and typically represents the rate at which the infected population changes. However, in discrete time models (18), the incidence is considered over a time period, which may represent the average generation interval (see “Generation Interval and Serial Interval”). If the generation interval is sufficiently small, then the definitions of incidence in continuous and discrete time models converge. Due to the discrepancy in these definitions (4), it has been suggested that, in continuous models, referring to the measure as “instantaneous incidence” may be more appropriate than incidence.

Disease prevalence is defined as the number of cases of a disease at a single time point in a population. In compartmental models, the prevalence is represented by the number of infectious individuals at any time.

### The Basic Reproduction Number (*R*_0_)

The basic reproduction number is defined as the average number of secondary cases caused by a single infectious individual in a totally susceptible population (19). This quantity is often used in deterministic compartmental models to determine whether an epidemic (in a demographic-free model) or endemic (in a model with demographics) will occur (*R*_0_ > 1) or if the disease will go extinct before causing an epidemic or endemic (*R*_0_ < 1). The case of *R*_0_ = 1 is referred to as a disease endemic, in which each case generates on average one additional case (20).

While there is a broad agreement on the definition of the reproduction number, recent studies show that the methodology and type of model used to calculate *R*_0_ may lead to different estimates (21, 22). Furthermore, variability in these estimates has raised concern about whether *R*_0_ should be used to determine the occurrence of an epidemic or endemic (21). In the context of simple susceptible-infectious-recovered (SIR) models, *R*_0_ is often derived from a relationship between the transmission rate and the infectious period (23). The simplest SIR epidemic model without demographics can be expressed by (13)

$$\begin{array}{l} \frac{\text{dS}}{\text{dt}}=-\text{incidence}=-\frac{\text{βSI}}{\text{N}}\\ \frac{\text{dI}}{\text{dt}}=\text{incidence}-\text{recovery}=\frac{\text{βSI}}{N}-\mathrm{\gamma I}\\ \frac{\text{dR}}{\text{dt}}=\text{recovery}=\mathrm{\gamma I} \end{array}$$

where $\frac{\text{d(}\cdot \text{)}}{\text{dt}}$ represents the change in a variable over time, *N* = *S* + *I* + *R* is the total population size, β is the transmission rate, and γ is the recovery rate. In this model, the inverse of the recovery rate (1/γ) represents the average length of the infectious period. At the early stages of an epidemic, the number of infected individuals is small compared to the number of susceptible individuals, which approximates the total population size and therefore *S*/*N* ≈ 1. This approximation simplifies the equation for the prevalence to

$$\frac{\text{dI}}{\text{dt}}=\mathrm{\beta I}-\mathrm{\gamma I}$$

Defining the quantity *R*_0_ = β/γ as the reproduction number (13) and solving the prevalence equation gives the solution $I\left(t\right)=I_{0}e^{\gamma (R_{0}-\;1)t}$, where *I*_0_ is the initial number of infections at the onset of the epidemic. This solution provides an approximation for the exponential growth of the prevalence at the early stages of an epidemic when *R*_0_ > 1. In order to illustrate the relationship between *R*_0_, the prevalence, and the disease incidence, we rewrite the prevalence in the form of *I*′ = γ*R*_0_*I* − γ*I*, and add this equation to the equation for recovery, which gives the incidence by *I*′ + *R*′ = γ*R*_0_*I*. This leads to the relation (24): incidence = γ*R*_0_ (prevalence), and therefore,

$$R_{0}=\left(\frac{\text{incidence}}{\text{prevalence}}\right)D$$

where *D* = 1/γ.

### Presymptomatic and Asymptomatic

The term “presymptomatic” describes a stage of disease in which an infected individual is infectious and can transmit the disease without presenting with symptoms. Individuals in the presymptomatic stage will proceed to become symptomatic at some point of time in the future. In contrast, asymptomatic refers to a disease stage in which individuals are infectious but are not exhibiting disease symptoms.

For example, influenza has various stages, including an asymptomatic stage in which individuals are capable of transmitting the disease but may have no knowledge of being infectious as they are not symptomatic. If these individuals develop a symptomatic infection, then the time period before the onset of symptoms is referred to as presymptomatic. A schematic representation of disease stages is illustrated in Figure 1.

### Generation Interval and Serial Interval

In modeling, the generation interval refers to the period of time between the onset of the infectious period in a primary case to the onset of the infectious period in a secondary case infected by the primary case (25). In epidemiology, the serial interval is defined as the period of time between the onset of symptoms in a primary case to the onset of symptoms in a secondary case infected by the primary case (26). The generation interval is not an observable period based on symptoms since an infected individual may become infectious and transmit the disease before symptoms appear. However, the serial interval is an observable period that is determined with the onset of symptoms. When the infectious period starts with the onset of symptoms (i.e., the latent and incubation periods have the same duration), the generation interval coincides with the serial interval.

The use of these terms in modeling is disease-specific and depends on the transmission characteristics of the pathogen. For example, in influenza, the onset of infectiousness and symptoms may differ in an infected person, and therefore, the duration of the serial interval and the generation interval may not be the same. Using these measures, as has been done in published studies (25, 27), to estimate some of the key epidemiological parameters, such as the reproduction number, may result in different outcomes in terms of transmissibility of the disease as well as different recommendations for public health interventions. However, since models are often based on the infectious period and not the onset of symptoms, it may be more appropriate to use the generation interval for understanding the transmission dynamics and estimating related parameters such as the basic reproduction number (28).

### Individual Immunity and Herd Immunity

Immunity refers to protection against a disease. It can be acquired naturally in an individual by experiencing the disease and recovering from infection or by other means such as active vaccination or passive transfer of maternal antibody across the placenta (29, 30). Herd immunity refers to the protection level of a population as a result of individuals’ immunity against infection. Immunity in individuals (involving innate and adaptive T and B cell immune responses) can provide a wide range of protection from full (which completely prevents the occurrence of infection temporarily or permanently) to partial (which may not prevent infection but may reduce its severity and mitigate outcomes). The degree of an individual’s immunity determines their susceptibility to infection or reinfection. Immunity can be partial or full, as previously stated, based on host or pathogen characteristics, and can wane over time. Compartmental transmission dynamic models often include the protection effects of immunity by averaging the levels of immune protection in individuals. It is, however, important to note that this average may not correspond to the potential disease transmission or disease outcomes at the individual level. Studies in immuno-epidemiology models are addressing these issues (31, 32).

### Attack Rate

The attack rate describes the proportion of the population that becomes infected over a specified period of time. For diseases for which not all infected individuals develop symptoms (e.g., influenza), it may be more informative to calculate the “clinical attack rate” (which measures the proportion of the population that develops disease symptoms as a result of an infection).

### Vaccine Efficacy and Effectiveness

In epidemiological and clinical studies, vaccine efficacy refers to the percentage reduction in the attack rate of the vaccinated cohort compared to the unvaccinated cohort as observed in a randomized controlled (field) trial. Vaccine effectiveness refers to the ability of a vaccine to prevent infection or related outcomes in the population in real-world conditions.

The inclusion of vaccine-induced immunity in disease dynamic models is generally governed by the reduction of disease transmission to vaccinated individuals. This reduction is often based on a parameter that quantifies vaccine efficacy or effectiveness. While these two terms have different meanings and are measured using distinct methods (33), many models have used them interchangeably to imply the average protection level of individuals in the population. It is important to clearly distinguish between the two terms of efficacy and effectiveness, and models should consider vaccine effectiveness in the study of disease transmission dynamics in the population.

While neither vaccine efficacy nor effectiveness is measured with respect to time, the effect of a vaccine in individuals may change over time. This is reflected in the waning of vaccine-induced immunity at the individual level and, as a result, the decline of herd immunity in the population. While there are various ways of including waning immunity in a model, it is important to note that a decline of immunity at the individual level over time is not equivalent to a decrease in vaccine efficacy or effectiveness.

## Discussion

Infectious disease modeling is an important epidemiological tool to inform strategies for disease control and prevention. Tracing its historical roots from the pioneering work of Daniel Bernoulli on smallpox in the 1760s (34) to the classical compartmental approach of Kermack and McKendrick in the 1920s (35, 36), modeling has evolved to incorporate demographic, geographic, and individual level characteristics, in addition to the most current knowledge of epidemiology, immunology, vaccines and drugs, and other public health interventions. This evolution is typified by advanced modeling and computational technologies, including metapopulation (37, 38), network (39), and agent-based simulation models (40, 41). From an epidemiological perspective, there are two important considerations that underlie the use of modeling tools, including disease progression-related terms (i.e., asymptomatic, presymptomatic, and symptomatic) and disease transmission-related terms (i.e., latent, infectious, and non-infectious) (12). Given the complexity of the newer generation of models that comes with their flexibility, a standardized lexicon of terms can play an important role in understanding their outcomes and establishing effective communication within and between disciplines involved in infectious disease management.

While a number of studies exist that aim to clarify and define modeling terms and methodologies in the context of infectious diseases (3, 4, 6, 7) to the best of our knowledge, this is the first attempt to develop a glossary of terms through the expertise of a virtual community of practice. This community allowed for an increased accessibility and participation by international participants, as well as easy and convenient access to the ongoing discussions. This initiative serves a larger goal envisioned for mod4PH, that is, to develop an international capacity and unified infrastructure that is capable of informing complex decision-making and improving health practices through the use of quality data, evidence, and scientific knowledge. The diversity of expertise in this community can help identify appropriate methods and modeling tools that can be used to assess the results and their comparability, share information on how models can best be used to inform policy, and enhance knowledge generation and translational activities in public and population health. Furthermore, this interdisciplinary nexus of constructive dialog can expand the training and research capacity beyond the traditional boundaries in academics and can enable consensus building and use of knowledge in a wider scientific community and international audience.

The diversity of the individuals in a community of practice does not come without its limitations. Various disciplines and backgrounds often use terms differently, and the lack of a common jargon-free language makes the process and intragroup discussions especially challenging. Furthermore, technical barriers to online forums (e.g., restrictions on the number of characters per post) may limit the extent of discussion that can take place in the platform. To improve communication between the mod4PH members, we implemented a convergent discussion process, enabling the discussion topics to remain active following their initiation in the online forum. This form of interdisciplinary engagement will ensure that, as our knowledge and understanding of infectious disease mechanisms enhances, discussion topics are improved and definitions of pertinent terms and their use in modeling efforts are adapted and informed.

The resulting glossary of terms from the current initiative of mod4PH is published as an online resource by the NCCID (10). As the usage and definition of the terms discussed here evolve, the online glossary will be updated to mirror the necessary changes and ensure that it remains a current reference for infectious disease modeling as an important tool that is increasingly applied to effectively respond to public health threats. This initiative is concordant with a key element of the 2016 “World Health Organization Research and Development Blueprint” for infectious diseases with epidemic potential (42), which calls for the international community to invest to improve its collective ability to respond to new threats and to prepare itself with a novel research and development paradigm to address future epidemics of newly emerging or re-emerging diseases.

While this study serves to bridge the gaps in modeling efforts and forge strong links between theory, policy, and practice, there remain many terms for ongoing discussion toward standardizing terminology, which are integral to the application of modeling outcomes to policy decision-making. We hope to use this study as a starting point to foster bidirectional communication between the involved disciplines, and improve the utility of modeling as an invaluable tool in the fight against persistent and emerging infectious diseases.

## Authors Note

The authors write for mod4PH (Modeling for Public Health).

## Author Contributions

RM and SM conceived the study and wrote the first draft of the manuscript. AS, JA, JH, AH, DS, EG, MH-B, HI-K, and JL contributed to the discussion, materials, and reviewing the paper. All authors contributed to the revisions and final version and accepted.

## Conflict of Interest Statement

The authors declare that the research was conducted in the absence of any commercial or financial relationships that could be construed as a potential conflict of interest.
